# IS YOUR COMMUNITY-BASED HEALTH PROMOTION PROGRAM EVIDENCE BASED AND READY FOR DISSEMINATION? HERE’S HOW TO FIND OUT

**DOI:** 10.1093/geroni/igz038.306

**Published:** 2019-11-08

**Authors:** Ellen C Schneider, Lesley Steinman, Casey Dicocco

**Affiliations:** 1 University of North Carolina at Chapel Hill, Chapel Hill, North Carolina, United States; 2 University of Washington Health Promotion Research Center, Seattle, Washington, United States; 3 U.S. Department of Health and Human Services, Administration on Aging/Administration for Community Living, Boston, Massachusetts, United States

## Abstract

Evidence-based programs (EBPs) offer proven ways to promote health and prevent disease among older adults in their communities. EBPs are based on rigorous study of the effects of specific interventions or model programs, demonstrate consistently positive changes in important health-related and functional measures, and have tools in place to maintain program access, quality, and efficiency across diverse settings. The University of North Carolina at Chapel Hill’s Center for Health Promotion and Disease Prevention (UNC HPDP), in partnership with the Evidence-Based Leadership Collaborative (EBLC), has established a review process and Review Council to identify new community programs that meet the evidence-based program criteria established by the Administration for Community Living (ACL), one of the chief U.S. federal agencies responsible for aging programs. Approved programs are then eligible for Older Americans Act Title III-D and other discretionary funding to support organizations that deliver EBPs to improve older adult health. The review process assesses the effectiveness, outcomes, and evaluation of the program, information about program implementation, training, and other key elements for successful program dissemination. The Review Council consists of national leaders with expertise in program research, evaluation, and implementation. The review process is supported by the ACL-funded National Chronic Disease Self-Management Education and Falls Prevention Resource Centers. This session will describe the ACL evidence-based health promotion program criteria that must be met for approval; an overview of the review process; and how researchers can submit their programs for review. Time will be allowed for questions, discussion, and research to practice implications.

